# A redetermination of 2-nitro­benzoic acid

**DOI:** 10.1107/S1600536809011830

**Published:** 2009-04-02

**Authors:** Gustavo Portalone

**Affiliations:** a Chemistry Department, "Sapienza" University of Rome, P.le A. Moro, 5, I-00185 Rome, Italy

## Abstract

The crystal structure of the title compound, C_7_H_5_NO_4_, was first reported by Kurahashi, Fukuyo & Shimada [(1967). *Bull. Chem. Soc. Jpn*, **40**, 1296]. It has been re-examined, improving the precision of the derived geometric parameters. The asymmetric unit comprises a non-planar independent mol­ecule, as the nitro and the carb­oxy substituents force each other to be twisted away from the plane of the aromatic ring by 54.9 (2) and 24.0 (2)°, respectively. The mol­ecules form a conventional dimeric unit *via* centrosymmetric inter­molecular hydrogen bonds.

## Related literature

For the previous structure determination, see: Kurahashi *et al.* (1967 ▶); Sakore *et al.* (1967 ▶); Tavale & Pant (1973 ▶). For the effect of nitro and carboxy substitution on the geometry of polysubstituted benzene rings, see: Colapietro *et al.* (1984 ▶); Domenicano *et al.* (1989 ▶). For the formation of hydrogen-bonded dimers in monocarboylic acids, see:Leiserowitz (1976 ▶). For computation of ring patterns formed by hydrogen bonds in crystal structures, see: Etter *et al.* (1990 ▶); Bernstein *et al.* (1995 ▶); Motherwell *et al.* (1999 ▶).
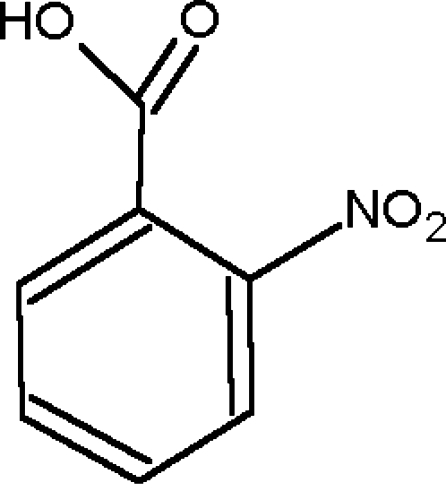


## Experimental

### 

#### Crystal data

C_7_H_5_NO_4_
*M* *_r_* = 167.12
Triclinic, 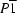

*a* = 5.0147 (15) Å
*b* = 7.527 (2) Å
*c* = 10.620 (2) Å
α = 69.41 (2)°
β = 86.07 (2)°
γ = 71.01 (3)°
*V* = 354.35 (18) Å^3^
*Z* = 2
Mo *K*α radiation
μ = 0.13 mm^−1^
*T* = 298 K
0.15 × 0.15 × 0.10 mm

#### Data collection

Oxford Diffraction Xcalibur S CCD diffractometer
Absorption correction: multi-scan (*CrysAlis RED*; Oxford Diffraction, 2006 ▶) *T*_min_ = 0.879, *T*_max_ = 0.980
3862 measured reflections
1872 independent reflections
1087 reflections with *I* > 2σ(*I*)
*R*_int_ = 0.050

#### Refinement

*R*[*F* ^2^ > 2σ(*F* ^2^)] = 0.077
*wR*(*F* ^2^) = 0.148
*S* = 1.07
1872 reflections
113 parameters
H atoms treated by a mixture of independent and constrained refinement
Δρ_max_ = 0.26 e Å^−3^
Δρ_min_ = −0.20 e Å^−3^

### 

Data collection: *CrysAlis CCD* (Oxford Diffraction, 2006 ▶); cell refinement: *CrysAlis RED* (Oxford Diffraction, 2006 ▶); data reduction: *CrysAlis RED*; program(s) used to solve structure: *SIR97* (Altomare *et al.*, 1999 ▶); program(s) used to refine structure: *SHELXL97* (Sheldrick, 2008 ▶); molecular graphics: *ORTEP-3* (Farrugia, 1997 ▶); software used to prepare material for publication: *WinGX* (Farrugia, 1999 ▶).

## Supplementary Material

Crystal structure: contains datablocks I, global. DOI: 10.1107/S1600536809011830/kp2213sup1.cif

Structure factors: contains datablocks I. DOI: 10.1107/S1600536809011830/kp2213Isup2.hkl

Additional supplementary materials:  crystallographic information; 3D view; checkCIF report

## Figures and Tables

**Table 1:** Hydrogen-bond geometry (Å, °)

*D*—H⋯*A*	*D*—H	H⋯*A*	*D*⋯*A*	*D*—H⋯*A*
O2—H2⋯O1^i^	0.90 (3)	1.77 (4)	2.660 (3)	173 (3)

Symmetry code: (i) 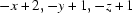.

**Fig. 1.**
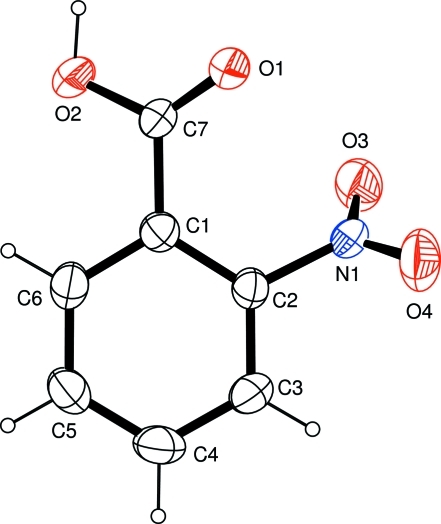
The molecular structure of (I), showing the atom-labelling scheme. Displacements ellipsoids are at the 50% probability level.

**Fig. 2.**
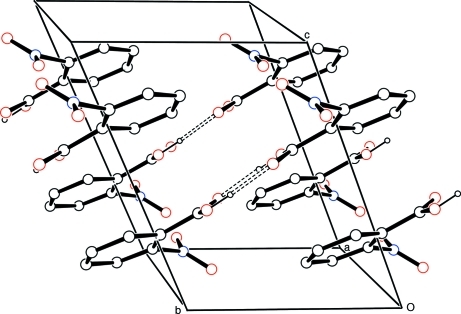
Crystal packing diagram for (I) viewed approximately down a. All atoms are shown as small spheres of arbitrary radii. For the sake of clarity, H atoms not involved in hydrogen bonding have been omitted. Hydrogen bonding is indicated by dashed lines.

## supplementary crystallographic information

### Comment

*o*-Nitrobenzoic acid was determined more than 40 years ago (Kurahashi *et al.*, 1967), but the final refinement was carried only to *R*=0.18. Subsequently, a new X-ray structure determination was reported (Sakore *et al.*, 1967). In this study, 1250 unique reflections were collected at ambient temperature on an equi-inclination Weissenberg camera using Cu Kα radiation. Data were corrected for Lp effects as well as for the effect of spot extension, but not for absorption [µ(Cu Kα)= 135 mm^-1^]. 694 visually estimated reflections having values significantly above background were used in the isotropic least-squares refinement. The final calculations led to *R* = 0.142 for 49 refined parameters, as the H atoms were not localized. A further anisotropic refinement of the structure was eventually carried out (Tavale & Pant, 1973). In this calculation, based on the same data set but with the inclusion of H atoms. the *R* factor decreased to 0.104, with a data-to-parameter ratio of 5.6, and average standard deviations of 0.013Å in C—C bond lengths and 0.9° in bond angles.

The asymmetric unit of (I) comprises a non-planar independent molecule, as the nitro and carboxy substituents force each other to be twisted away from the plane of the aromatic ring by 54.9 (2) and 24.0 (2)°, respectively (Fig. 1). The pattern of bond lengths and bond angles is consistent with those reported in previous structural investigations concerning the effect of the nitro and the carboxy groups on the geometry of polysubstituted benzene rings (Colapietro *et al.*, 1984; Domenicano *et al.*, 1989). Analysis of the crystal packing of (I), (Fig. 2), shows that the molecular components form the conventional dimeric units observed in monocarboylic acids (Leiserowitz, 1976). The structure is stabilized by very short [2.660 (3) Å] intermolecular O—H···O interactions of descriptor *R*^2^_2_(8) (Etter *et al.*, 1990; Bernstein *et al.*, 1995; Motherwell *et al.*, 1999) (Table 1) between the OH moiety and the carbonyl O atom (O1^i^) [symmetry code: (i) -*x* + 2, -*y* + 1, -*z* + 1].

### Experimental

*o*-Nitrobenzoic acid (0.1 mmol, Sigma Aldrich at 95% purity) was dissolved in water (5 ml) and gently heated under reflux for 1 h. After cooling the solution to an ambient temperature, crystals suitable for single-crystal X-ray diffraction were grown by slow evaporation of the solvent after few days.

### Refinement

All H atoms were found in a difference map and then treated as riding atoms, with C—H = 0.93Å and *U*_iso_ values equal to 1.2*U*_eq_(C, phenyl). The remaining H atom of the carboxy group was freely refined.

### Crystal data

**Table:**

C_7_H_5_NO_4_	*Z* = 2
*M**_r_* = 167.12	*F*(000) = 172
Triclinic, *P*1	*D*_x_ = 1.566 Mg m^−^^3^
Hall symbol: -P 1	Mo *K*α radiation, λ = 0.71073 Å
*a* = 5.0147 (15) Å	Cell parameters from 861 reflections
*b* = 7.527 (2) Å	θ = 3.0–32.5°
*c* = 10.620 (2) Å	µ = 0.13 mm^−^^1^
α = 69.41 (2)°	*T* = 298 K
β = 86.07 (2)°	Tablets, colourless
γ = 71.01 (3)°	0.15 × 0.15 × 0.10 mm
*V* = 354.35 (18) Å^3^	

### Data collection

**Table:**

Oxford Diffraction Xcalibur S CCD diffractometer	1872 independent reflections
Radiation source: Enhance (Mo) X-ray Source	1087 reflections with *I* > 2σ(*I*)
graphite	*R*_int_ = 0.050
Detector resolution: 16.0696 pixels mm^-1^	θ_max_ = 29.0°, θ_min_ = 3.0°
ω and φ scans	*h* = −6→6
Absorption correction: multi-scan (*CrysAlis RED*; Oxford Diffraction, 2006)	*k* = −10→10
*T*_min_ = 0.879, *T*_max_ = 0.980	*l* = −14→14
3862 measured reflections	

### Refinement

**Table:**

Refinement on *F*^2^	Primary atom site location: structure-invariant direct methods
Least-squares matrix: full	Secondary atom site location: difference Fourier map
*R*[*F*^2^ > 2σ(*F*^2^)] = 0.077	Hydrogen site location: inferred from neighbouring sites
*wR*(*F*^2^) = 0.148	H atoms treated by a mixture of independent and constrained refinement
*S* = 1.07	*w* = 1/[σ^2^(*F*_o_^2^) + (0.0536*P*)^2^ + 0.0451*P*] where *P* = (*F*_o_^2^ + 2*F*_c_^2^)/3
1872 reflections	(Δ/σ)_max_ < 0.001
113 parameters	Δρ_max_ = 0.26 e Å^−^^3^
0 restraints	Δρ_min_ = −0.20 e Å^−^^3^

### Special details

**Table:**

Experimental. Absorption correction: CrysAlis RED, Oxford Diffraction Ltd., Version 1.171.32.29 (release 10-06-2008 CrysAlis171 .NET) (compiled Jun 10 2008,16:49:55) Empirical absorption correction using spherical harmonics, implemented in SCALE3 ABSPACK scaling algorithm.
Geometry. All e.s.d.'s (except the e.s.d. in the dihedral angle between two l.s. planes) are estimated using the full covariance matrix. The cell e.s.d.'s are taken into account individually in the estimation of e.s.d.'s in distances, angles and torsion angles; correlations between e.s.d.'s in cell parameters are only used when they are defined by crystal symmetry. An approximate (isotropic) treatment of cell e.s.d.'s is used for estimating e.s.d.'s involving l.s. planes.
Refinement. Refinement of *F*^2^ against ALL reflections. The weighted *R*-factor *wR* and goodness of fit *S* are based on *F*^2^, conventional *R*-factors *R* are based on *F*, with *F* set to zero for negative *F*^2^. The threshold expression of *F*^2^ > σ(*F*^2^) is used only for calculating *R*-factors(gt) *etc*. and is not relevant to the choice of reflections for refinement. *R*-factors based on *F*^2^ are statistically about twice as large as those based on *F*, and *R*- factors based on ALL data will be even larger.

### Fractional atomic coordinates and isotropic or equivalent isotropic displacement parameters (Å^2^)

**Table:**

	*x*	*y*	*z*	*U*_iso_*/*U*_eq_	
O1	0.8083 (4)	0.3762 (3)	0.6058 (2)	0.0471 (6)	
O2	1.2483 (4)	0.2482 (3)	0.5512 (2)	0.0481 (6)	
H2	1.221 (7)	0.378 (5)	0.504 (3)	0.068 (11)*	
O3	0.7790 (4)	0.2147 (3)	0.8921 (2)	0.0537 (6)	
O4	0.4118 (4)	0.1514 (3)	0.8517 (2)	0.0533 (6)	
N1	0.6642 (5)	0.1288 (3)	0.8481 (2)	0.0344 (5)	
C1	1.0287 (5)	0.0235 (3)	0.6923 (2)	0.0305 (6)	
C2	0.8416 (5)	−0.0231 (4)	0.7933 (2)	0.0297 (6)	
C3	0.8224 (6)	−0.2123 (4)	0.8508 (3)	0.0381 (7)	
H3	0.6904	−0.2375	0.9147	0.046*	
C4	1.0025 (6)	−0.3648 (4)	0.8123 (3)	0.0450 (7)	
H4	0.9927	−0.4941	0.8503	0.054*	
C5	1.1971 (6)	−0.3249 (4)	0.7172 (3)	0.0440 (8)	
H5	1.3219	−0.4286	0.6933	0.053*	
C6	1.2084 (6)	−0.1327 (4)	0.6572 (3)	0.0393 (7)	
H6	1.3386	−0.1080	0.5922	0.047*	
C7	1.0188 (5)	0.2327 (4)	0.6144 (3)	0.0334 (6)	

### Atomic displacement parameters (Å^2^)

**Table:**

	*U*^11^	*U*^22^	*U*^33^	*U*^12^	*U*^13^	*U*^23^
O1	0.0457 (12)	0.0296 (10)	0.0542 (13)	−0.0083 (9)	0.0228 (10)	−0.0080 (9)
O2	0.0461 (13)	0.0350 (12)	0.0543 (13)	−0.0151 (9)	0.0236 (10)	−0.0071 (10)
O3	0.0600 (14)	0.0563 (13)	0.0657 (14)	−0.0290 (11)	0.0177 (11)	−0.0391 (12)
O4	0.0361 (12)	0.0638 (14)	0.0638 (15)	−0.0131 (10)	0.0160 (10)	−0.0321 (12)
N1	0.0411 (14)	0.0319 (12)	0.0275 (12)	−0.0140 (10)	0.0117 (10)	−0.0071 (10)
C1	0.0335 (14)	0.0287 (14)	0.0275 (13)	−0.0088 (11)	0.0023 (11)	−0.0090 (11)
C2	0.0312 (13)	0.0300 (14)	0.0270 (13)	−0.0084 (10)	0.0045 (11)	−0.0106 (11)
C3	0.0486 (17)	0.0355 (15)	0.0323 (15)	−0.0205 (12)	0.0114 (13)	−0.0097 (12)
C4	0.062 (2)	0.0263 (14)	0.0437 (17)	−0.0150 (13)	0.0056 (15)	−0.0079 (13)
C5	0.0530 (18)	0.0312 (15)	0.0436 (17)	−0.0052 (12)	0.0085 (14)	−0.0169 (13)
C6	0.0402 (16)	0.0393 (16)	0.0357 (15)	−0.0105 (12)	0.0132 (13)	−0.0138 (13)
C7	0.0392 (15)	0.0320 (14)	0.0304 (14)	−0.0148 (12)	0.0131 (12)	−0.0116 (11)

### Geometric parameters (Å, °)

**Table:**

O1—C7	1.222 (3)	C2—C3	1.372 (3)
O2—C7	1.310 (3)	C3—C4	1.380 (3)
O2—H2	0.90 (3)	C3—H3	0.9300
O3—N1	1.208 (3)	C4—C5	1.381 (4)
O4—N1	1.221 (3)	C4—H4	0.9300
N1—C2	1.474 (3)	C5—C6	1.379 (4)
C1—C6	1.381 (3)	C5—H5	0.9300
C1—C2	1.402 (3)	C6—H6	0.9300
C1—C7	1.485 (3)		
			
C7—O2—H2	108 (2)	C3—C4—C5	119.8 (2)
O3—N1—O4	124.6 (2)	C3—C4—H4	120.1
O3—N1—C2	118.2 (2)	C5—C4—H4	120.1
O4—N1—C2	117.1 (2)	C6—C5—C4	120.6 (2)
C6—C1—C2	117.2 (2)	C6—C5—H5	119.7
C6—C1—C7	119.9 (2)	C4—C5—H5	119.7
C2—C1—C7	122.7 (2)	C5—C6—C1	120.9 (2)
C3—C2—C1	122.5 (2)	C5—C6—H6	119.6
C3—C2—N1	116.4 (2)	C1—C6—H6	119.6
C1—C2—N1	121.1 (2)	O1—C7—O2	123.4 (2)
C2—C3—C4	118.9 (2)	O1—C7—C1	122.2 (2)
C2—C3—H3	120.6	O2—C7—C1	114.4 (2)
C4—C3—H3	120.6		
			
C6—C1—C2—C3	−3.7 (4)	C2—C3—C4—C5	−0.1 (4)
C7—C1—C2—C3	169.9 (3)	C3—C4—C5—C6	−1.9 (4)
C6—C1—C2—N1	173.2 (2)	C4—C5—C6—C1	1.1 (4)
C7—C1—C2—N1	−13.3 (4)	C2—C1—C6—C5	1.6 (4)
O3—N1—C2—C3	122.6 (3)	C7—C1—C6—C5	−172.1 (3)
O4—N1—C2—C3	−53.9 (3)	C6—C1—C7—O1	152.9 (3)
O3—N1—C2—C1	−54.5 (3)	C2—C1—C7—O1	−20.5 (4)
O4—N1—C2—C1	129.1 (3)	C6—C1—C7—O2	−23.9 (4)
C1—C2—C3—C4	2.9 (4)	C2—C1—C7—O2	162.7 (2)
N1—C2—C3—C4	−174.1 (2)		

### Hydrogen-bond geometry (Å, °)

**Table:**

*D*—H···*A*	*D*—H	H···*A*	*D*···*A*	*D*—H···*A*
O2—H2···O1^i^	0.90 (3)	1.77 (4)	2.660 (3)	173 (3)

Symmetry codes: (i) −*x*+2, −*y*+1, −*z*+1.
